# Mechanotransduction and Cytoskeleton Remodeling Shaping YAP1 in Gastric Tumorigenesis

**DOI:** 10.3390/ijms20071576

**Published:** 2019-03-29

**Authors:** Jinglin Zhang, Yuhang Zhou, Patrick M.K. Tang, Alfred S.L. Cheng, Jun Yu, Ka Fai To, Wei Kang

**Affiliations:** 1 Department of Anatomical and Cellular Pathology, State Key Laboratory of Translational Oncology, Prince of Wales Hospital, The Chinese University of Hong Kong, Shatin, Hong Kong, China; zhangjinglin987@gmail.com (J.Z.); zyhjoe@gmail.com (Y.Z.); patrick.tang@cuhk.edu.hk (P.M.K.T.); 2 Institute of Digestive Disease, State Key Laboratory of Digestive Disease, The Chinese University of Hong Kong, Shatin, Hong Kong, China; junyu@cuhk.edu.hk; 3 Li Ka Shing Institute of Health Science, Sir Y.K. Pao Cancer Center, The Chinese University of Hong Kong, Shatin, Hong Kong, China; 4 School of Biomedical Sciences, The Chinese University of Hong Kong, Shatin, Hong Kong, China; alfredcheng@cuhk.edu.hk; 5 Department of Medicine and Therapeutics, The Chinese University of Hong Kong, Shatin, Hong Kong, China

**Keywords:** Hippo pathway, YAP1, mechanical strain, F-actin, gastric cancer

## Abstract

The essential role of Hippo signaling pathway in cancer development has been elucidated by recent studies. In the gastrointestinal tissues, deregulation of the Hippo pathway is one of the most important driving events for tumorigenesis. It is widely known that Yes-associated protein 1 (YAP1) and WW domain that contain transcription regulator 1 (TAZ), two transcriptional co-activators with a PDZ-binding motif, function as critical effectors negatively regulated by the Hippo pathway. Previous studies indicate the involvement of YAP1/TAZ in mechanotransduction by crosstalking with the extracellular matrix (ECM) and the F-actin cytoskeleton associated signaling network. In gastric cancer (GC), YAP1/TAZ functions as an oncogene and transcriptionally promotes tumor formation by cooperating with TEAD transcription factors. Apart from the classic role of Hippo-YAP1 cascade, in this review, we summarize the current investigations to highlight the prominent role of YAP1/TAZ as a mechanical sensor and responder under mechanical stress and address its potential prognostic and therapeutic value in GC.

## 1. Introduction

According to the report of GLOBOCAN 2018, gastric cancer (GC) is the fifth most diagnosed and the third most life-threatening cancer globally. Over one billion new cases and 783,000 death cases are reported this year. In Eastern Asian countries and regions, including China, South Korea and Japan, the incidence is much higher than other countries [1]. The risk factors of GC mainly are *Helicobacter pylori* (*H. pylori*) and Epstein-Barr virus (EBV) infection, smoking, chronic gastritis with glandular atrophy, intestinal metaplasia, and, most importantly, genetic alterations. Unhealthy life styles including high-salt diet, low-vegetable diet, and alcohol intake are strongly associated with GC development [2]. Symptoms of GC are not easily perceived in early stages, directly leading to its poor prognosis.

Gastric adenocarcinoma accounts for the majority of the GC cases. It is histologically classified as intestinal and diffuse type. Based on molecular characteristics, it is also defined as four subtypes, namely EBV-infected tumors, microsatellite instability (MSI) tumors, genomically stable (GS) tumors, and chromosomally instable (CIN) tumors, by The Cancer Genome Atlas (TCGA) project [3]. GC is considered as a multistage disease with complicated genetic and epigenetic alterations [4]. Herein, the changes of oncogenic factors, tumor suppressors, mismatch repair factors, cell adhesion related molecules and cell-cycle regulators contribute to tumor growth and metastasis [5]. A previous study pointed out that CDH17 and APOE mRNAs were correlated with invasion of tumor cells, as well as the lymph node metastasis. FUS, COL1A1, COL1A2, and APOE were also found to be associated with advanced GCs [6]. Another earlier study indicated that twelve highly methylated genes were found to be associated with GC progression [7]. In the last few years, other groups identified a subset of tumor suppresser genes [8] and microRNAs [9] with unique expression patterns in different GC stages and subtypes. These dysregulated genes were considered as prognostic biomarkers, which might serve as druggable targets for intervention therapy. Recently, multiple signaling pathways, such as Wnt/β-catenin, Notch, nuclear factor-κB (NFκB), Sonic Hedgehog (Shh), and epidermal growth factor receptor (EGFR) signaling pathways have been shown to play oncogenic roles in gastric carcinogenesis. These aberrantly activated signaling pathways are deemed to be prominent targets for specific antibodies or small molecules [10]. Among them, Hippo signaling pathway emerges as an important regulator involved in kinds of fundamental physiological development processes and multiple tumor progressions [11]. The signaling transduction of Hippo pathway is regulated by the physical and mechanical context between the cells and the surrounding extracellular matrix [12,13]. It is also known that mechanical mechanisms also promote tumor growth through activating cell proliferation, differentiation and some other cellular events [14,15,16]. In GC, how the mechanotransduction contributes to GC progression has not been comprehensively elucidated. In the present review, we aim to summarize the recent mechanical findings in Hippo pathway and try to reveal its distinguishing role in GC development.

## 2. The Emerging Role of Hippo Pathway in Solid Tumors Especially in GC

### 2.1. Main Components of the Hippo Cascade

Mammalian Hippo signaling pathway is evolutionarily conserved and composed of several kinases, mammalian Ste20-like kinases 1 and 2 (MST1/2), and Large tumor suppressor kinase 1 and 2 (LATS1/2) [17]. MST1/2 phosphorylates LATS1/2 and MOB1 (Mobkl1a/b), leading to their activation [18]. The activation of these components are essential for organ size control and tumor growth inhibition [19,20]. Activated LATS1/2 further phosphorylates Yes-associated protein 1 (YAP1) [20] and WW domain that contain transcription regulator 1 (TAZ), which are two oncogenic paralogs related to cancer initiation and development [21]. The phosphorylation of YAP1/TAZ leads its association with 14-3-3, thus YAP1/TAZ is quenched in the cytoplasm. Consequently, its nuclear translocation to promote tumorigenesis is sequestered. Otherwise, the unphosphorylated YAP1 and TAZ will be translocated into the nucleus and bind with TEA domain DNA-binding transcription factors (TEADs), to promote cell proliferation and differentiation [22,23,24]. The key components of Hippo cascade constitute a phosphokinase axis to negatively regulate the downstream effectors to maintain homeostasis and prevent tumor growth [25]. Additionally, some studies demonstrated that YAP1 can also prevent p73 from ubiquitination mediated by Itch [26], thus driving apoptosis and cell death as an outcome of DNA damage [27]. The p73/YAP1 association directly activates the transcription of Promyelocytic leukemia (PML). In turn, PML also interacts with the specific domains of YAP1 to keep its abundance [28]. Hence, apart from cytoplasmic phosphorylation and degradation, YAP1 also exerts anti-tumor effect by inducing apoptosis, which is Hippo pathway independent.

YAP1, also known as Yes-associated protein (YAP65), is a proline-rich modular adaptor initially identified by its binding affinity with the SRC homology 3 (SH3) domain of non-receptor tyrosine kinase c-Yes [29]. It serves as the most crucial transcriptional regulator of Hippo signaling pathway. The activation of YAP1 can be also mediated by mechanical strain. To respond to the mechanical stimulation, activated YAP1 interplays with multiple signaling pathways and triggers various cellular events, including cell proliferation, migration [30], and differentiation [31]. Regarding the development of GC, YAP1 exerts oncogenic role by interacting with TEAD1 and TEAD4 transcription factors [32]. However, the upstream regulators of YAP1 and signaling networks of mechanical stress are unknown. Thus, we here review the recent studies and try to depict how YAP1 senses and responds to the mechanical signals and its potential role in cytoskeleton remodeling in solid tumors including gastric adenocarcinoma.

### 2.2. Other Factors Participating in Hippo Signaling Pathway

Accumulating evidences demonstrate that the tumor suppressive role of Hippo signaling pathway is also crosstalking with multiple factors and other signaling pathways. Salvador Homolog 1 (SAV1) is a scaffold protein for MST1/2. The phosphorylation of SAV1 facilitates the activation of LATS1/2 [33]. In epithelial cells, E-cadherin is proposed to interact with α-catenin and β-catenin to form a complex, which activates the downstream Hippo signaling pathway to limit the cell growth [34,35]. Similarly, Angiomotin (AMOT) family proteins are important upstream components in Hippo signaling that directly interact with YAP1 and confine its subcellular localization [36]. The LKB1-MARK signaling is demonstrated as a required upstream regulator, which enhances the activation of Hippo signaling pathway and keep YAP1/TAZ from nuclear translocation [37]. Table 1 summarizes several important components involved in Hippo pathway.

### 2.3. Dysregulated YAP1 in Gastric Tumorigenicity

The Hippo kinases signaling determines the subcellular localization and nuclear accumulation of YAP1 and TAZ. Breaking the Hippo kinase signaling increases the nuclear translocation of YAP1 or TAZ. It is well known that YAP and TAZ are transcriptional co-regulators that bind with TEADs to form a DNA-binding complex. Dysregulated expression of YAP1 has been reported to promote tumor progression. The abundance of YAP1 in the cytoplasm and nucleus was first described in some specific types of GC [38]. Recently, in vitro and in vivo studies clearly demonstrated the aberrant activation of YAP/TAZ not only drives tumor growth but also is associated with tumor metastasis [39,40]. Intriguingly, under biomechanical cues, cell mobility is driven only by YAP1 but not by TAZ. Although TAZ shares 46% amino acid sequence and similar functional domain with YAP1, it has very distinctive functions [41]. One piece of evidence of this is that TAZ lacks hydrophobic linker sequences in TEAD-binding region [42].

To unravel the underlying mechanism of the oncogenic role of YAP1–TEAD axis, molecular characteristics of YAP1 have been identified. YAP1 is located in Chromosome 11q22 with a recurrent amplification partner cIAP1 in multiple carcinomas, including esophageal squamous cell carcinoma [43], liver cancer [44], a subset of cervical tumors [45] and lung carcinomas [46]. The specific interaction of YAP1 with other binding partners are mediated by its WW domain. Through the WW domain, the expression of proliferation-related genes is transcriptionally activated. PDZ-binding motif of YAP1 is another functional domain mainly responsible for its nuclear translocation, which facilitates the tumorigenesis by activating the related gene expression [47]. Considering that YAP1 has cytoplasmic-nuclear shuttling property, a latest report presents that SET1A-mediated mono-methylation at K342 of YAP1 promotes its accumulation in the nuclei, which serves as a novel mechanism to elucidate how YAP1 nuclear translocation facilitates tumorigenesis [48]. Meanwhile, some important findings point out that multiple oncogenic signaling pathways are crosstalking with YAP1 mediated cascade, including Wnt/β-catenin [49], Notch [50], SRC tyrosine kinase, and transforming growth factor beta (TGF-β) signaling pathways. In β-catenin-driving tumorigenesis, YAP1 forms a complex with β-catenin and a transcription factor TBX5. This complex antagonizes cell apoptosis and enhances cell survival [51]. Moreover, another report indicates the β-catenin/TCF4 complex upregulates YAP1 expression in colorectal cancer (CRC) cells via binding to the promoter region of YAP1 [52]. The crosstalk between YAP1 and Notch signaling has also been highlighted in previous studies. YAP1 upregulates both ligand [53] and receptor [54] of Notch signaling pathway from the transcriptional level. Apart from the canonical Hippo pathway, integrin-SRC tyrosine kinase signaling pathway is another dominant regulator for YAP1 [55]. In squamous epithelium cells, integrins are upstream activators of SRC. Activated SRC drives YAP1 to transfer into the nucleus of the basal layer cells thus to activate the transcription of genes for regenerating or tumor progression. Suppressing this oncogenic signaling might serve as a novel strategy to maintain epithelial homeostasis and inhibit tumor growth [56,57]. SRC phosphorylates and actives YAP1 at three sites to link YAP1 with the tyrosine kinase signaling. The impact of Hippo-YAP1 signaling on TGF-β signaling depends on the subcellular localization and activation status of YAP1/TAZ. Cytoplasmic YAP1/TAZ interacts with SMAD proteins to attenuate the TGF-β induced transformation, while nuclear YAP1/TAZ can also binds with SMAD proteins to augment the stimulatory effects of TGF-β [58,59].

YAP1 has been shown as an initiator and driver and it is highly upregulated in GC patients to indicate poor outcomes [60,61]. The YAP1 nuclear accumulation indicates a worse disease specific survival (DSS). In MKN45, which is a GC cell line with YAP1 homozygous deletion. Ectopic expression of YAP1 induces significant increased abilities of cell proliferation and monolayer colony formation. Invasive phenotypes after YAP1 overexpression were observed in vitro and in vivo [62]. Meanwhile, knocking down YAP1 in GC cell line inhibits the cell proliferation, migration and invasion abilities, suggesting its prominent oncogenic role in GC progression [63]. With the oncogenic role of YAP1 gradually revealed in GC, YAP1 related molecular mechanisms gained extensive attention. As a transcription co-activator, YAP1 directly activates MYC, CTGF and AXL expression. It has been accepted that deregulated MYC critically leads to cell transformation and tumor progression [64]. Activated MYC has been reported to affect early-stage gastric carcinogenesis [65]. Previous studies indicate MYC is a direct downstream mediator of YAP1 [66]. The significant role of YAP1 in initiating gastric carcinogenesis by upregulating MYC has also been addressed [60]. In addition to MYC, the role of YAP1 in gastric carcinogenesis and metastasis also found through cooperating with other adaptor proteins. In GC, overexpressed YAP1 positively correlates with survivin protein expression [67]. Meanwhile, YAP1 promotes the oncogenic transformation by directly interacting with RUNX2 and suppressing p21 expression [68]. It can also exerts promoting property through downstream effector, receptor tyrosine kinase AXL [69]. We previously demonstrated that YAP1’s transformation ability is required for constitutive activation of MAPK/ERK signaling cascade [62], which is known as a key signaling cascade regulating cell survival and cell differentiation [70]. Multiple miRNAs have been reported to be involved in GC as tumor suppressors and some of them directly target YAP1. We identified that YAP1 is negatively regulated by miR-15a and miR-16-1, and YAP1/TEAD4-CTGF axis components were co-targeted by miR-375 [71,72]. Dysfunction of these tumor suppressive miRNAs results in YAP1 overexpression and subsequent tumorigenesis.

## 3. Mechanical Signal Transduction Involved in Hippo Pathway in Cancer Cells

Mechanical and physical forces are widely existing in the cell–cell contacts and cell microenvironment, regulating multiple physiological events, such as cell proliferation, migration, differentiation, and cell death [73,74]. To understand how these factors affect cells and tissues, much attention has been paid to the cytoskeleton rearrangement and the signal transduction in cells [75]. Mechanical mechanisms are indispensable for tissue generation, and they also exert critical functional roles in tumorigenesis [76,77,78]. How they play such distinctive roles is still under investigation. Apart from the fundamental role of Hippo signaling in development and tumor growth, increasing studies indicate the nuclear YAP1 serves as an effective sensor and responder for mechanical stimulus [79]. Under mechanical context, YAP1 coordinates with E-cadherin, β-Catenin [80], and MYC [66] and integrates multiple signaling pathways to contribute to tumor growth. Here, we summarize the major biomechanical signals and their reciprocal interaction with YAP1.

### 3.1. Stiffness in Tumor Microenvironment

Unlike normal tissues, the tumor microenvironment consists of reconstituted extracellular matrix (ECM), blood vasculature, immune cells, and supporting stromal cells [81]. The increased collagens and other small molecules are commonly found in tumor microenvironment [82]. The interactions of these components provide various chemical signal transductions and intensive physical outputs, which enhances the matrix stiffness in the microenvironment [83]. Increased matrix stiffness is positively correlated with malignancy status in many types of solid tumors. The matrix can crosstalk with Rho/ERK signaling, and focal adhesion (FA) assembly [76]. The localization and activity of YAP1 is constantly regulated by mechanical signals such as cell attachment and ECM. Different from non-tumorous tissues, the reciprocal interaction of YAP1 and substrate rigidity induces genetic, epigenetic and phenotypic changes during tumor progression [84,85]. Mechanical activation of YAP1 leads to uncontrollable growth [85] and metastatic phenotypes of cancer cells [86]. In breast cancer, stiff matrix is found to be associated with poor survival and tumor aggressiveness [87], probably due to the activation of MYC and the suppression of phosphatase and tensin homolog (PTEN) and homeobox A9 (HOXA9) [88]. In endothelial cells, stiffness promotes tumor metastasis via activating the CCN1/CYR61/β-catenin/N-cadherin cascade and enhancing the binding affinity of the tumor cells to the endothelium [89]. CCN1/CYR61 is also noted as a famous downstream target of YAP1 in cancers, contributing to tumor growth [90]. In epithelial cells, matrix stiffness also promotes epithelial–mesenchymal transition (EMT) by activating YAP1 through mechanotransduction pathways. In turn, the activated YAP1 also increases the expression of metalloproteinases to reinforce ECM stiffness [91]. The positive feedback loop of YAP1 and ECM composition not only maintains tumor growth and metastasis, but also antagonizes chemotherapy [92,93]. The situation is quite different in low stiffness matrix condition. Nuclear translocation of YAP1 is attenuated and its oncogenic function is quenched [85]. Most adult epithelial cells are existing in soft substrate with relatively weak proliferative ability. These cells only express extremely low YAP1, and the activation form of YAP1 is quite limited. Tumor development is significantly suppressed in such cells [94]. The regulatory mechanisms are gradually revealed recently. Meng et al. identified that Ras-related GTPase RAP2 was activated under soft stiffness through PDZGEF1 and PDZGEF2. RAP2 stimulates Rho GTPase Activating Protein 29 (ARHGAP29) and MAP4K4, -6, and -7 to phosphorylate MST1/2 and LATS1/2. The phosphorylated LATS1/2 further suppresses YAP1/TAZ activation. RAP2 was identified as a key switch mediating the mechanotransduction pathway to link matrix stiffness to the transcriptional activities [95]. The function of YAP1 was previously shown to be blocked by the interaction with SWI/SNF in the nucleus. ARID1A mediates the formation of this complex. Because of the interaction with SWI/SNF, nuclear YAP1 is unable to bind with TEADs. Moreover, this interaction is related to the actin dynamics. In low stiffness matrix, the formation of filament actin (F-actin) fibers are inhibited, which facilitates the ARID1A-SWI/SNF-YAP1 axis to play functional roles against tumorigenesis [96]. However, Hippo pathway is not involved in ARID1A-SWI/SNF-YAP1 axis.

YAP1 activation plays a critical role in driving tumorigenesis, however how YAP1 interplays with the ECM stiffness to promote GC remains elusive. According to the current studies, rigidity-driven tumor invasion is actin-cytoskeleton and integrin dependent. The actin cytoskeleton has physical connection with the ECM or neighbor cells by means of the FA complex. Integrins interact with the FA complex to link the external forces with actin cytoskeleton [97]. Further, actin filaments and their associated FA complexes act as mechanosensors to transduce the strength of external forces along the actin filaments network into biochemical signaling. This signal transduction rises a series of cellular responses including cell migration to harmonize the cell status and the surrounding ECM, and facilitate metastasis [98,99,100,101,102]. YAP1 has been shown as an essential sensor and responder between tumor cells and their microenvironment. To unravel the comprehensive role of Hippo pathway in matrix stiffness mediated tumorigenesis, the molecular network of actin cytoskeleton regulation and YAP1 localization remains to be further investigation.

### 3.2. Cell Density

In addition to the stiffness generated by matrix substrates, the cell density also exerts mechanical stimulation on cellular activities. Hippo signaling pathway is well-recognized as one of the major pathways correlated to cell density [11]. In adult mammalian epithelial cells, the growth ability is impaired under high cell density, which is known as cell–cell contact inhibition. The transcription-promoting role of YAP1 is inhibited under high cell density, mainly due to the activation of the upstream tumor suppressive kinases [103]. In malignant tumor, cells continuously grow by resisting contact inhibition and the Hippo signaling is found dysfunctional. Overexpressed YAP1 and nuclear accumulated YAP1 in turn regulate gene expression to promote cell proliferation [20,104]. Overcoming contact inhibition is one of the distinctive properties of malignant transformation in cancer cells. How malignant cells sense and respond to the density changes is still under investigation and discussion [105]. Downregulated miRNAs may partially contribute to the accumulation of YAP1. For an instance, a cell density sensitive miRNA named miR-590-5p was shown down-regulated in GC cells and its binding site at YAP1 3’UTR was identified in colorectal cancer [106,107]. Hopefully, illustrating the aberrant activation mechanisms of YAP1 in malignant cells may unveil the cell contact influence on Hippo pathway.

Although the malignant cells can bypass cell contact inhibition, cancer cells are still sensitive to cell density. It has been identified that common cancer cell lines show less propensity of vascular invasion in vitro and metastasis in vivo under high density, while the invasiveness is augmented in a low cell density situation. Regard to this finding, the expression level of E-cadherin is upregulated and Hippo pathway is activated in high density condition. However, low cell density decreases E-cadherin, but increases cytokine secretion and nuclear YAP1 accumulation [108]. Cell density increases actin polymerization except for the cells with undetectable YAP1 expression. In GC cells, a recent report indicates that ARHGAP29, a downstream target of YAP1, destabilizes F-actin into globular actin (G-actin) by suppressing a RhoA dependent pathway [109]. Taken together, YAP1 is highly expressed and activated in GC cells, promoting cell proliferation to breakthrough contact inhibition. In addition, YAP1 abundance also benefits vascular invasion by triggering malignant transformation and cytoskeleton rearrangement.

### 3.3. Shear Stress

Shear stress is also one of the mechanical forces in the lumen of lymphatics and vessels, affecting the circulating cancer cell and immune cell adhesion. The flow force influences multiple characters of solid tumor cells, including the surrounding concentrations of cytokines and chemokines, transportation of tumor antigens and chemo-agents [110]. How flow forces affect intracellular activities in tumors have not been fully identified. In a recent study, lymphatic fluid guides transportation of aggressive tumor cells towards lymph nodes and facilitates metastasis [41]. This study verified that the expression of 36 genes was altered by the activation of YAP1 under shear stress. Most of these genes are previously found contributing to cell migration, invasion, adhesion, angiogenesis, and lamellipodia/filopodia formation by in vitro and in vivo studies [111,112,113]. Although the functional role of shear stress in tumor growth and metastasis has not been fully clarified, the activation of YAP1 may be considered as a key point for understanding the comprehensive mechanism and identifying novel therapeutic strategies.

## 4. Cytoskeleton Remodeling Regulates YAP1 Localization in Solid Tumors Including GC

Mechanical cues from microenvironment manipulate cell growth and metastasis mainly through the cytoskeleton remodeling [114]. Hippo signaling displays a dominant role in modulating cytoskeleton changes. It crosstalks with related signaling pathways as well as some surface receptors and cytoplasmic proteins [79]. Located in the cell membrane, tight and adherent junctional proteins are upstream activators of Hippo pathway and negatively regulate YAP1 nuclear translocation [12,80]. A recent study demonstrated that increase of ECM density leads to the dissociation of the membranous E-cadherin/β-catenin complex. The released β-catenin is translocated into the nucleus to cooperate with YAP1 in triggering GC cell proliferation, EMT, and chemotherapy resistance. Moreover, it was proposed that cell-ECM interaction relies on integrin signaling and phosphorylation of focal adhesion kinases (FAK), while cell–cell adhesion requires the E-cadherin/β-catenin complex [115]. Focal adhesions (FAs) are dynamic membrane structures that perceive mechanical signals and bridge the integrin-ECM and cytoskeleton [116]. The Hippo signaling pathway was shown to be modulated by the integrin-FA cascade [117]. FAK mediates the SRC activation, which subsequently counteracts the phosphorylation of LATS and increases nuclear accumulation of YAP1. PI3K-PDK1 exerts important role in this regulatory axis. In tumorigenesis, the functional role of SRC in regulating YAP1 was validated by a recent study [118]. The integrin-SRC signaling plays a dominant role in recruiting YAP1 into the nuclei. It is reported that the Enigma family proteins interacts with F-actin polymers in Integrin-FAs cascade to promote YAP1 activation by SRC kinases under mechanical stretch [119]. To some extent, this finding fills the gap between integrin-FA-SRC cascade and the nuclear translocation of YAP1 in mechanotransduction. However, more evidence is still needed to verify these regulatory mechanisms during gastric tumorigenesis.

G-protein coupled receptors (GPCRs) cascade also contribute significant role in regulating the actin dynamics and cytoskeleton rearrangement in response to the mechanical stress. GPCRs can differentially control the Hippo signaling pathway by promoting or blocking of actin polymerization, which is the central regulator of Hippo-YAP1 pathway [120,121]. GPCRs bind to Gα12/13, Gαq/11, and Gαi/o to transduce extracellular signals through Rho activation and actin polymerization. This cascade induces YAP1 activation by inhibiting LATS kinases. On the opposite, GPCRs can also bind with Gαs to activate the cAMP-PKA signaling pathway. This reinforces the effect of Hippo kinases and quench YAP1 in the cytoplasm [122]. The effect of GPCRs on YAP1 nuclear translocation might be Hippo kinase independent as well, although the evidence is insufficient currently [123]. As an important upstream regulator of the Hippo-YAP1 pathway, it is necessary to investigate the potential involvement of GPCR signaling in the GC progression.

As mentioned by previous studies, YAP1 initiates gastric carcinogenesis. It also functions as a mechanical sensor and responder in many kinds of solid tumors. The alteration of cytoskeleton and cytoskeleton-associated proteins enable cancer cells to resist chemotherapy and facilitate its metastasis to distant sites [124,125]. Elucidating the underlying functional roles of these proteins may shed light on novel therapeutic strategies. Rho family GTPases are small G proteins that connect membrane receptors and the associated signaling processes, including Integrin-FA-SRC signaling, GPCR-G protein signaling, and actin cytoskeleton rearrangement [126]. Rho family GTPases are required for tumor transformation, proliferation, invasion, metastasis, and angiogenesis [127,128,129]. The mRNA expression of several Rho-GTPase members was found significantly upregulated in GC patients and cell lines [130]. RhoA, Rac1 and Cdc42 are the three major family members of these upregulated Rho-GTPases [131]. They control the activity of YAP1 via a LATS kinase independent manner and regulate actin dynamics in cancer cells [13,79]. An early study indicated that RhoA/ROCK signaling pathway facilitates the invasion ability of scirrhous gastric carcinoma cells by increasing the activity of Rac [132]. Similarly, gain of function mutation of RhoA was identified in poor prognosis diffuse type of GC [133]. Rho-GTPases obviously play significant roles in gastric carcinogenesis. In addition, the dynamics of actin cytoskeleton are dominantly regulated by Rho-GTPases. Rho stimulates the assembly of contractile actin stress fibers by triggering downstream effectors, while Rac and Cdc42 promote the of F-actin networks [134]. Rho is considered to be a stronger and more stringent regulator for YAP/TAZ activity than Rac and Cdc42 [135]. Meanwhile, inhibiting ROCK also obstructs the nuclear translocation of YAP1 [85]. Evidence indicates F-actin cytoskeleton intermediates the mechanical signals and the YAP1 activity. In general, the reorganization of F-actin includes polymerization and depolymerization. These two processes engage in morphologic transformation to increase motility of the cancer cells [136]. In our previous study, we observed disorganization of F-actin and reduced EMT in GC cell lines when knocking down the Slit-Robo GTPase-activating protein (SRGAP1), which is a GAP for Rho-GTPases [137]. In colorectal cancer, RASAL2, which encodes a RAS-GTPase-activating protein (RAS-GAP), functions as an independent factor for prognosis. This study also demonstrated the oncogenic role of RASAL2 by targeting LATS2/YAP1 axis of Hippo pathway [138]. The stability of F-actin also correlates with YAP1. As reported, actin capping protein CAP-Z and severing proteins Cofilin and Gelsolin work coordinately to stabilize the F-actin architecture and modulate YAP nuclear translocation [120]. Moreover, suppression of the RhoA-LIMK-Cofilin pathway leads to depolymerization of F-actin and decreases the GC cell motility. This report provides new insight into the functional role of YAP in metastasis by regulating actin dynamics [109].

**Figure 1:**
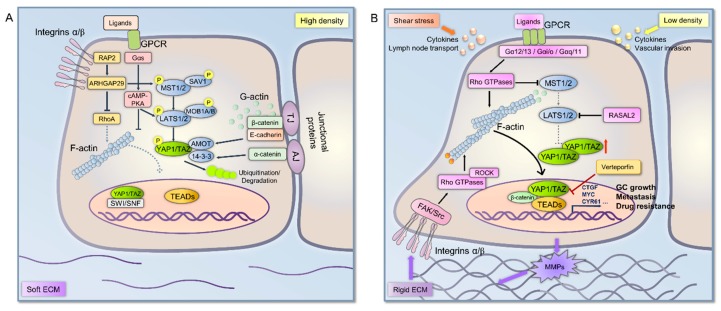
Proposed mechanical cues regulates Hippo signaling pathway in gastric tumorigenesis. (**A**) Hippo pathway turn on and YAP1 inactivation under soft ECM or high cell density. Cells reside in soft ECM and ECM exerts low mechanical force on gastric cells. Integrin signaling is inactivated. RAP2/ARHGAP29 axis activates MST1/2 and LATS1/2, as well as other Hippo pathway components. The GPCR/Gαs directly phosphorylates LATS1/2 via a cAMP/PKA dependent manner. Mechanical force also triggers SWI/SNF activation in the nucleus to bind with YAP1, preventing the interaction between YAP1 and TEADs. Meanwhile, under high cell density, E-cadherin/β-catenin and α-catenin bind on the junctional proteins, mediating AMOT and 14-3-3 to quench YAP1. Due to the activation of Hippo pathway, YAP1/TAZ is retained in the cytoplasm and undergoes further degradation. Transcription activity of TEADs is thus suppressed. Moreover, the activation of RhoA and formation of F-actin fibers are abrogated by ARHGAP29 in a Hippo pathway independent manner. (**B**) Hippo pathway turn off and YAP1 activation under rigid ECM, low cell density, and shear stress. Rigid ECM causes the activation of integrin signaling and promotes the assembly of FA/SRC complex. Rho-GTPases facilitate the polymerization of F-actin cytoskeleton. F-actin guides the nuclear translocation of YAP1/TAZ. Besides, ligands bind to GPCRs to recruit downstream G proteins, Gα12/13, Gαq/11, and Gαi/o. Through Rho-GTPases, GPCR signaling suppresses the functional role of Hippo pathway and increase YAP1/TAZ nuclear translocation. Overexpressed YAP1/TAZ and the downstream effectors promote GC cell grow, metastasis, and drug resistance. Production of MMPs are increased, further enhancing ECM rigidity. Low cell density enables the cytokine secretion which is required for vascular invasion. Shear stress also leads to the accumulation of cytokines for metastasis and lymph nodes transportation. Verteporfin has been proved as a prominent small molecule targeting YAP1-TEAD and inhibiting tumorigenicity. ECM, extracellular matrix; FA, focal adhesion; TJ, tight junction; AJ, adherent junction; MMP, matrix metalloproteinases.

Based on the latest reports, the overall regulation of mechanical signaling and cytoskeleton rearrangement on YAP1 has been proposed, as shown in Figure 1.

## 5. Conclusions and Future Directions

Hippo signaling pathway is necessary to maintain the homeostasis of gastric and other gastrointestinal organs. Deregulation of Hippo kinases is associated with tumorigenicity including gastric adenocarcinoma through hyperactivation of YAP1/TAZ. Apart from that, emerging microenvironment factors, intracellular components and the structure of cytoskeleton also impact on the translocation of YAP1 through a mechanical transduction mechanism. This point has attracted more and more research interests. In GC, the tumor thickness and increased stiffness of tumor surrounding wall exert mechanical inputs on the microenvironment and cells. This will lead to the inactivation of Hippo pathway to activate YAP1/TAZ subsequently.

Activating the Hippo pathway is considered to be a promising anti-tumor therapeutic strategy. Suppressing YAP1-TEAD activity is the main philosophy. However, drugs targeting YAP1 are still limited in number and the efficacies are not yet satisfactory. Verteporfin (VP) is a small-molecule inhibitor of YAP1 that directly disrupts the YAP1-TEAD interaction in mammals. VP has been shown to inhibit cell growth in vitro, but its anti-tumor efficacy needs further clinical verification [139]. More importantly, since Hippo signaling is a central hub of various signaling pathways, targeting the related crosstalked signaling pathways as combinational therapy is a preferable strategy. For example, GPCRs and G proteins function as key transducers of extracellular molecules which regulate Hippo pathway. Activating GPCR-cAMP-PKA signaling can reinforce Hippo pathway to suppress YAP1-TEAD activity. In primary GC samples, mutation of GPCRs are frequently detected in advance-stage tumors. In this regard, the GPCR-Hippo signaling crosstalk might be proposed as a promising therapeutic target [140].

Nevertheless, the Hippo-YAP1 signaling pathway is just part of the regulatory framework in gastric tumorigenicity. Several issues need to be addressed in the subsequent studies. Firstly, both tissue regeneration and carcinogenesis require the suppression of Hippo pathway and activation of YAP1/TAZ. The detailed mechanisms of these two processes have not been differentially clarified. Likely, potential positive feedback mechanisms are involved in GC to ensure the continuous nuclear accumulation of YAP1. Secondly, based on different rigidity of substrates, cancer cells develop specific migration modes during metastasis. Thus, tumor cells in different GC subtypes may behave differently. How YAP1/TAZ modulates the cell property and behavior in this process has not been fully determined. Thirdly, the Hippo pathway crosstalks with multiple signaling pathways that are relevant to cell growth, transformation and metastasis. However, little is known about the accurate epigenetic regulation mechanism involved in the microenvironment shaping YAP1. Hopefully, deciphering the functional role of YAP1 in the microenvironment shaping process and revealing how YAP1 interplays with intracellular factors or ECM components to promote tumorigenesis may help to identify more diagnostic markers and therapeutic targets for clinical translation.

## Figures and Tables

**Table 1:** Main components and properties of Hippo pathway.

Core Components	Properties	References
MST1/2	Activated by cellular stressors and represent tumor suppressor function	[17]
SAV1	A scaffold protein of MST1/2. Phosphorylation of SAV1 activates of LATS1/2	[33]
MOB1	Binds to MST2 and activates LATS1	[18]
LATS1/2	Activated by MST1/2 and represents tumor suppressor function	[17]
AMOT	Directly interacts with YAP1 and confines its nuclear translocation	[36]
YAP1	Negative downstream effector of Hippo pathway and plays an oncogenic role in driving tumorigenesis	[20]
TAZ1	A paralog of YAP1 and shares similar function of YAP1	[21]
TEAD1-4	Transcription factors and cooperate with YAP1/TAZ	[22,23,24]

## References

[1] Bray F., Ferlay J., Soerjomataram I., Siegel R.L., Torre L.A., Jemal A. (2018). Global cancer statistics 2018: GLOBOCAN estimates of incidence and mortality worldwide for 36 cancers in 185 countries. CA Cancer J. Clin..

[2] Uemura N., Okamoto S., Yamamoto S., Matsumura N., Yamaguchi S., Yamakido M., Taniyama K., Sasaki N., Schlemper R.J. (2001). Helicobacter pylori infection and the development of gastric cancer. N. Engl. J. Med..

[3] Cancer Genome Atlas Research Network (2014). Comprehensive molecular characterization of gastric adenocarcinoma. Nature.

[4] Correa P. (1992). Human gastric carcinogenesis: A multistep and multifactorial process—First American Cancer Society Award Lecture on Cancer Epidemiology and Prevention. Cancer Res..

[5] Yasui W., Oue N., Aung P.P., Matsumura S., Shutoh M., Nakayama H. (2005). Molecular-pathological prognostic factors of gastric cancer: A review. Gastric Cancer.

[6] Oue N., Hamai Y., Mitani Y., Matsumura S., Oshimo Y., Aung P.P., Kuraoka K., Nakayama H., Yasui W. (2004). Gene expression profile of gastric carcinoma: Identification of genes and tags potentially involved in invasion, metastasis, and carcinogenesis by serial analysis of gene expression. Cancer Res..

[7] Oue N., Mitani Y., Motoshita J., Matsumura S., Yoshida K., Kuniyasu H., Nakayama H., Yasui W. (2006). Accumulation of DNA methylation is associated with tumor stage in gastric cancer. Cancer.

[8] Lee H.S., Lee H.K., Kim H.S., Yang H.K., Kim W.H. (2003). Tumour suppressor gene expression correlates with gastric cancer prognosis. J. Pathol..

[9] Li X., Zhang Y., Zhang Y., Ding J., Wu K., Fan D. (2010). Survival prediction of gastric cancer by a seven-microRNA signature. Gut.

[10] Wu W.K., Cho C.H., Lee C.W., Fan D., Wu K., Yu J., Sung J.J. (2010). Dysregulation of cellular signaling in gastric cancer. Cancer Lett..

[11] Pan D. (2010). The hippo signaling pathway in development and cancer. Dev. Cell.

[12] Schroeder M.C., Halder G. (2012). Regulation of the Hippo pathway by cell architecture and mechanical signals. Semin. Cell Dev. Biol..

[13] Seo J., Kim J. (2018). Regulation of Hippo signaling by actin remodeling. BMB Rep..

[14] Quaglino A., Salierno M., Pellegrotti J., Rubinstein N., Kordon E.C. (2009). Mechanical strain induces involution-associated events in mammary epithelial cells. BMC Cell Biol..

[15] Altman G.H., Horan R.L., Martin I., Farhadi J., Stark P.R., Volloch V., Richmond J.C., Vunjak-Novakovic G., Kaplan D.L. (2002). Cell differentiation by mechanical stress. FASEB J..

[16] Chaturvedi L.S., Marsh H.M., Basson M.D. (2007). Src and focal adhesion kinase mediate mechanical strain-induced proliferation and ERK1/2 phosphorylation in human H441 pulmonary epithelial cells. Am. J. Physiol. Cell Physiol..

[17] Chan E.H., Nousiainen M., Chalamalasetty R.B., Schafer A., Nigg E.A., Sillje H.H. (2005). The Ste20-like kinase Mst2 activates the human large tumor suppressor kinase Lats1. Oncogene.

[18] Praskova M., Xia F., Avruch J. (2008). MOBKL1A/MOBKL1B phosphorylation by MST1 and MST2 inhibits cell proliferation. Curr. Biol..

[19] Zeng Q., Hong W. (2008). The emerging role of the hippo pathway in cell contact inhibition, organ size control, and cancer development in mammals. Cancer Cell.

[20] Zhao B., Wei X., Li W., Udan R.S., Yang Q., Kim J., Xie J., Ikenoue T., Yu J., Li L. (2007). Inactivation of YAP oncoprotein by the Hippo pathway is involved in cell contact inhibition and tissue growth control. Genes Dev..

[21] Lei Q.Y., Zhang H., Zhao B., Zha Z.Y., Bai F., Pei X.H., Zhao S., Xiong Y., Guan K.L. (2008). TAZ promotes cell proliferation and epithelial-mesenchymal transition and is inhibited by the hippo pathway. Mol. Cell. Biol..

[22] Wu S., Liu Y., Zheng Y., Dong J., Pan D. (2008). The TEAD/TEF family protein Scalloped mediates transcriptional output of the Hippo growth-regulatory pathway. Dev. Cell.

[23] Zhao B., Ye X., Yu J., Li L., Li W., Li S., Yu J., Lin J.D., Wang C.Y., Chinnaiyan A.M. (2008). TEAD mediates YAP-dependent gene induction and growth control. Genes Dev..

[24] Vassilev A., Kaneko K.J., Shu H., Zhao Y., DePamphilis M.L. (2001). TEAD/TEF transcription factors utilize the activation domain of YAP65, a Src/Yes-associated protein localized in the cytoplasm. Genes Dev..

[25] Zhao B., Tumaneng K., Guan K.L. (2011). The Hippo pathway in organ size control, tissue regeneration and stem cell self-renewal. Nat. Cell Biol..

[26] Levy D., Adamovich Y., Reuven N., Shaul Y. (2007). The Yes-associated protein 1 stabilizes p73 by preventing Itch-mediated ubiquitination of p73. Cell Death Differ..

[27] Strano S., Monti O., Pediconi N., Baccarini A., Fontemaggi G., Lapi E., Mantovani F., Damalas A., Citro G., Sacchi A. (2005). The transcriptional coactivator Yes-associated protein drives p73 gene-target specificity in response to DNA Damage. Mol. Cell.

[28] Lapi E., Di Agostino S., Donzelli S., Gal H., Domany E., Rechavi G., Pandolfi P.P., Givol D., Strano S., Lu X. (2008). PML, YAP, and p73 are components of a proapoptotic autoregulatory feedback loop. Mol. Cell.

[29] Sudol M. (1994). Yes-associated protein (YAP65) is a proline-rich phosphoprotein that binds to the SH3 domain of the Yes proto-oncogene product. Oncogene.

[30] Yang X.M., Cao X.Y., He P., Li J., Feng M.X., Zhang Y.L., Zhang X.L., Wang Y.H., Yang Q., Zhu L. (2018). Overexpression of Rac GTPase Activating Protein 1 Contributes to Proliferation of Cancer Cells by Reducing Hippo Signaling to Promote Cytokinesis. Gastroenterology.

[31] Lange A.W., Sridharan A., Xu Y., Stripp B.R., Perl A.K., Whitsett J.A. (2015). Hippo/Yap signaling controls epithelial progenitor cell proliferation and differentiation in the embryonic and adult lung. J. Mol. Cell Biol..

[32] Zhou Y., Huang T., Zhang J., Wong C.C., Zhang B., Dong Y., Wu F., Tong J.H.M., Wu W.K.K., Cheng A.S.L. (2017). TEAD1/4 exerts oncogenic role and is negatively regulated by miR-4269 in gastric tumorigenesis. Oncogene.

[33] Tapon N., Harvey K.F., Bell D.W., Wahrer D.C., Schiripo T.A., Haber D., Hariharan I.K. (2002). salvador Promotes both cell cycle exit and apoptosis in Drosophila and is mutated in human cancer cell lines. Cell.

[34] Kim N.G., Koh E., Chen X., Gumbiner B.M. (2011). E-cadherin mediates contact inhibition of proliferation through Hippo signaling-pathway components. Proc. Natl. Acad. Sci. USA.

[35] Schlegelmilch K., Mohseni M., Kirak O., Pruszak J., Rodriguez J.R., Zhou D., Kreger B.T., Vasioukhin V., Avruch J., Brummelkamp T.R. (2011). Yap1 acts downstream of alpha-catenin to control epidermal proliferation. Cell.

[36] Zhao B., Li L., Lu Q., Wang L.H., Liu C.Y., Lei Q., Guan K.L. (2011). Angiomotin is a novel Hippo pathway component that inhibits YAP oncoprotein. Genes Dev..

[37] Mohseni M., Sun J., Lau A., Curtis S., Goldsmith J., Fox V.L., Wei C., Frazier M., Samson O., Wong K.K. (2014). A genetic screen identifies an LKB1-MARK signalling axis controlling the Hippo-YAP pathway. Nat. Cell Biol..

[38] Lam-Himlin D.M., Daniels J.A., Gayyed M.F., Dong J., Maitra A., Pan D., Montgomery E.A., Anders R.A. (2006). The hippo pathway in human upper gastrointestinal dysplasia and carcinoma: A novel oncogenic pathway. Int. J. Gastrointest. Cancer.

[39] Galli G.G., Carrara M., Yuan W.C., Valdes-Quezada C., Gurung B., Pepe-Mooney B., Zhang T., Geeven G., Gray N.S., de Laat W. (2015). YAP Drives Growth by Controlling Transcriptional Pause Release from Dynamic Enhancers. Mol. Cell.

[40] Stein C., Bardet A.F., Roma G., Bergling S., Clay I., Ruchti A., Agarinis C., Schmelzle T., Bouwmeester T., Schubeler D. (2015). YAP1 Exerts Its Transcriptional Control via TEAD-Mediated Activation of Enhancers. PLoS Genet..

[41] Lee H.J., Diaz M.F., Price K.M., Ozuna J.A., Zhang S., Sevick-Muraca E.M., Hagan J.P., Wenzel P.L. (2017). Fluid shear stress activates YAP1 to promote cancer cell motility. Nat. Commun..

[42] Guo X., Zhao B. (2013). Integration of mechanical and chemical signals by YAP and TAZ transcription coactivators. Cell Biosci..

[43] Imoto I., Yang Z.Q., Pimkhaokham A., Tsuda H., Shimada Y., Imamura M., Ohki M., Inazawa J. (2001). Identification of cIAP1 as a candidate target gene within an amplicon at 11q22 in esophageal squamous cell carcinomas. Cancer Res..

[44] Zender L., Spector M.S., Xue W., Flemming P., Cordon-Cardo C., Silke J., Fan S.T., Luk J.M., Wigler M., Hannon G.J. (2006). Identification and validation of oncogenes in liver cancer using an integrative oncogenomic approach. Cell.

[45] Imoto I., Tsuda H., Hirasawa A., Miura M., Sakamoto M., Hirohashi S., Inazawa J. (2002). Expression of cIAP1, a target for 11q22 amplification, correlates with resistance of cervical cancers to radiotherapy. Cancer Res..

[46] Dai Z., Zhu W.G., Morrison C.D., Brena R.M., Smiraglia D.J., Raval A., Wu Y.Z., Rush L.J., Ross P., Molina J.R. (2003). A comprehensive search for DNA amplification in lung cancer identifies inhibitors of apoptosis cIAP1 and cIAP2 as candidate oncogenes. Hum. Mol. Genet..

[47] Oka T., Remue E., Meerschaert K., Vanloo B., Boucherie C., Gfeller D., Bader G.D., Sidhu S.S., Vandekerckhove J., Gettemans J. (2010). Functional complexes between YAP2 and ZO-2 are PDZ domain-dependent, and regulate YAP2 nuclear localization and signalling. Biochem. J..

[48] Fang L., Teng H., Wang Y., Liao G., Weng L., Li Y., Wang X., Jin J., Jiao C., Chen L. (2018). SET1A-Mediated Mono-Methylation at K342 Regulates YAP Activation by Blocking Its Nuclear Export and Promotes Tumorigenesis. Cancer Cell.

[49] Varelas X., Miller B.W., Sopko R., Song S., Gregorieff A., Fellouse F.A., Sakuma R., Pawson T., Hunziker W., McNeill H. (2010). The Hippo pathway regulates Wnt/β-catenin signaling. Dev. Cell.

[50] Slemmons K.K., Crose L.E.S., Riedel S., Sushnitha M., Belyea B., Linardic C.M. (2017). A Novel Notch-YAP Circuit Drives Stemness and Tumorigenesis in Embryonal Rhabdomyosarcoma. Mol. Cancer Res..

[51] Rosenbluh J., Nijhawan D., Cox A.G., Li X., Neal J.T., Schafer E.J., Zack T.I., Wang X., Tsherniak A., Schinzel A.C. (2012). β-Catenin-driven cancers require a YAP1 transcriptional complex for survival and tumorigenesis. Cell.

[52] Konsavage W.M., Kyler S.L., Rennoll S.A., Jin G., Yochum G.S. (2012). Wnt/β-catenin signaling regulates Yes-associated protein (YAP) gene expression in colorectal carcinoma cells. J. Biol. Chem..

[53] Tschaharganeh D.F., Chen X., Latzko P., Malz M., Gaida M.M., Felix K., Ladu S., Singer S., Pinna F., Gretz N. (2013). Yes-associated protein up-regulates Jagged-1 and activates the Notch pathway in human hepatocellular carcinoma. Gastroenterology.

[54] Yimlamai D., Christodoulou C., Galli G.G., Yanger K., Pepe-Mooney B., Gurung B., Shrestha K., Cahan P., Stanger B.Z., Camargo F.D. (2014). Hippo pathway activity influences liver cell fate. Cell.

[55] Li P., Silvis M.R., Honaker Y., Lien W.H., Arron S.T., Vasioukhin V. (2016). alphaE-catenin inhibits a Src-YAP1 oncogenic module that couples tyrosine kinases and the effector of Hippo signaling pathway. Genes Dev..

[56] Elbediwy A., Vincent-Mistiaen Z.I., Spencer-Dene B., Stone R.K., Boeing S., Wculek S.K., Cordero J., Tan E.H., Ridgway R., Brunton V.G. (2016). Integrin signalling regulates YAP and TAZ to control skin homeostasis. Development.

[57] Taniguchi K., Wu L.W., Grivennikov S.I., de Jong P.R., Lian I., Yu F.X., Wang K., Ho S.B., Boland B.S., Chang J.T. (2015). A gp130-Src-YAP module links inflammation to epithelial regeneration. Nature.

[58] Ferrigno O., Lallemand F., Verrecchia F., L’Hoste S., Camonis J., Atfi A., Mauviel A. (2002). Yes-associated protein (YAP65) interacts with Smad7 and potentiates its inhibitory activity against TGF-β/Smad signaling. Oncogene.

[59] Hiemer S.E., Szymaniak A.D., Varelas X. (2014). The transcriptional regulators TAZ and YAP direct transforming growth factor β-induced tumorigenic phenotypes in breast cancer cells. J. Biol. Chem..

[60] Choi W., Kim J., Park J., Lee D.H., Hwang D., Kim J.H., Ashktorab H., Smoot D., Kim S.Y., Choi C. (2018). YAP/TAZ Initiates Gastric Tumorigenesis via Upregulation of MYC. Cancer Res..

[61] Yu L., Gao C., Feng B., Wang L., Tian X., Wang H., Ma D. (2017). Distinct prognostic values of YAP1 in gastric cancer. Tumour Biol..

[62] Kang W., Tong J.H., Chan A.W., Lee T.L., Lung R.W., Leung P.P., So K.K., Wu K., Fan D., Yu J. (2011). Yes-associated protein 1 exhibits oncogenic property in gastric cancer and its nuclear accumulation associates with poor prognosis. Clin. Cancer Res..

[63] Sun D., Li X., He Y., Li W., Wang Y., Wang H., Jiang S., Xin Y. (2016). YAP1 enhances cell proliferation, migration, and invasion of gastric cancer in vitro and in vivo. Oncotarget.

[64] Chung H.J., Levens D. (2005). c-myc expression: Keep the noise down!. Mol. Cells.

[65] Calcagno D.Q., Leal M.F., Assumpcao P.P., Smith M.A., Burbano R.R. (2008). MYC and gastric adenocarcinoma carcinogenesis. World J. Gastroenterol..

[66] Croci O., De Fazio S., Biagioni F., Donato E., Caganova M., Curti L., Doni M., Sberna S., Aldeghi D., Biancotto C. (2017). Transcriptional integration of mitogenic and mechanical signals by Myc and YAP. Genes Dev..

[67] Da C.L., Xin Y., Zhao J., Luo X.D. (2009). Significance and relationship between Yes-associated protein and survivin expression in gastric carcinoma and precancerous lesions. World J. Gastroenterol..

[68] Vitolo M.I., Anglin I.E., Mahoney W.M., Renoud K.J., Gartenhaus R.B., Bachman K.E., Passaniti A. (2007). The RUNX2 transcription factor cooperates with the YES-associated protein, YAP65, to promote cell transformation. Cancer Biol. Ther..

[69] Cui Z.L., Han F.F., Peng X.H., Chen X., Luan C.Y., Han R.C., Xu W.G., Guo X.J. (2012). YES-associated protein 1 promotes adenocarcinoma growth and metastasis through activation of the receptor tyrosine kinase Axl. Int. J. Immunopathol. Pharmacol..

[70] Roberts P.J., Der C.J. (2007). Targeting the Raf-MEK-ERK mitogen-activated protein kinase cascade for the treatment of cancer. Oncogene.

[71] Kang W., Huang T., Zhou Y., Zhang J., Lung R.W.M., Tong J.H.M., Chan A.W.H., Zhang B., Wong C.C., Wu F. (2018). miR-375 is involved in Hippo pathway by targeting YAP1/TEAD4-CTGF axis in gastric carcinogenesis. Cell Death Dis..

[72] Kang W., Tong J.H., Lung R.W., Dong Y., Zhao J., Liang Q., Zhang L., Pan Y., Yang W., Pang J.C. (2015). Targeting of YAP1 by microRNA-15a and microRNA-16-1 exerts tumor suppressor function in gastric adenocarcinoma. Mol. Cancer.

[73] Wei S.C., Yang J. (2016). Forcing through Tumor Metastasis: The Interplay between Tissue Rigidity and Epithelial-Mesenchymal Transition. Trends Cell Biol..

[74] Jiang L., Sun Z., Chen X., Li J., Xu Y., Zu Y., Hu J., Han D., Yang C. (2016). Cells Sensing Mechanical Cues: Stiffness Influences the Lifetime of Cell-Extracellular Matrix Interactions by Affecting the Loading Rate. ACS Nano.

[75] Provenzano P.P., Keely P.J. (2011). Mechanical signaling through the cytoskeleton regulates cell proliferation by coordinated focal adhesion and Rho GTPase signaling. J. Cell Sci..

[76] Paszek M.J., Zahir N., Johnson K.R., Lakins J.N., Rozenberg G.I., Gefen A., Reinhart-King C.A., Margulies S.S., Dembo M., Boettiger D. (2005). Tensional homeostasis and the malignant phenotype. Cancer Cell.

[77] Ulrich T.A., de Juan Pardo E.M., Kumar S. (2009). The mechanical rigidity of the extracellular matrix regulates the structure, motility, and proliferation of glioma cells. Cancer Res..

[78] Wozniak M.A., Desai R., Solski P.A., Der C.J., Keely P.J. (2003). ROCK-generated contractility regulates breast epithelial cell differentiation in response to the physical properties of a three-dimensional collagen matrix. J. Cell Biol..

[79] Low B.C., Pan C.Q., Shivashankar G.V., Bershadsky A., Sudol M., Sheetz M. (2014). YAP/TAZ as mechanosensors and mechanotransducers in regulating organ size and tumor growth. FEBS Lett..

[80] Benham-Pyle B.W., Pruitt B.L., Nelson W.J. (2015). Cell adhesion. Mechanical strain induces E-cadherin-dependent Yap1 and β-catenin activation to drive cell cycle entry. Science.

[81] Chaudhuri O., Koshy S.T., Branco da Cunha C., Shin J.W., Verbeke C.S., Allison K.H., Mooney D.J. (2014). Extracellular matrix stiffness and composition jointly regulate the induction of malignant phenotypes in mammary epithelium. Nat. Mater..

[82] Ng M.R., Brugge J.S. (2009). A stiff blow from the stroma: Collagen crosslinking drives tumor progression. Cancer Cell.

[83] Spill F., Reynolds D.S., Kamm R.D., Zaman M.H. (2016). Impact of the physical microenvironment on tumor progression and metastasis. Curr. Opin. Biotechnol..

[84] Halder G., Dupont S., Piccolo S. (2012). Transduction of mechanical and cytoskeletal cues by YAP and TAZ. Nat. Rev. Mol. Cell Biol..

[85] Dupont S., Morsut L., Aragona M., Enzo E., Giulitti S., Cordenonsi M., Zanconato F., Le Digabel J., Forcato M., Bicciato S. (2011). Role of YAP/TAZ in mechanotransduction. Nature.

[86] Lamar J.M., Stern P., Liu H., Schindler J.W., Jiang Z.G., Hynes R.O. (2012). The Hippo pathway target, YAP, promotes metastasis through its TEAD-interaction domain. Proc. Natl. Acad. Sci. USA.

[87] Seewaldt V. (2014). ECM stiffness paves the way for tumor cells. Nat. Med..

[88] Mouw J.K., Yui Y., Damiano L., Bainer R.O., Lakins J.N., Acerbi I., Ou G., Wijekoon A.C., Levental K.R., Gilbert P.M. (2014). Tissue mechanics modulate microRNA-dependent PTEN expression to regulate malignant progression. Nat. Med..

[89] Reid S.E., Kay E.J., Neilson L.J., Henze A.T., Serneels J., McGhee E.J., Dhayade S., Nixon C., Mackey J.B., Santi A. (2017). Tumor matrix stiffness promotes metastatic cancer cell interaction with the endothelium. EMBO J..

[90] Zanconato F., Forcato M., Battilana G., Azzolin L., Quaranta E., Bodega B., Rosato A., Bicciato S., Cordenonsi M., Piccolo S. (2015). Genome-wide association between YAP/TAZ/TEAD and AP-1 at enhancers drives oncogenic growth. Nat. Cell Biol..

[91] Wei S.C., Fattet L., Tsai J.H., Guo Y., Pai V.H., Majeski H.E., Chen A.C., Sah R.L., Taylor S.S., Engler A.J. (2015). Matrix stiffness drives epithelial-mesenchymal transition and tumour metastasis through a TWIST1-G3BP2 mechanotransduction pathway. Nat. Cell Biol..

[92] Calvo F., Ege N., Grande-Garcia A., Hooper S., Jenkins R.P., Chaudhry S.I., Harrington K., Williamson P., Moeendarbary E., Charras G. (2013). Mechanotransduction and YAP-dependent matrix remodelling is required for the generation and maintenance of cancer-associated fibroblasts. Nat. Cell Biol..

[93] Rice A.J., Cortes E., Lachowski D., Cheung B.C.H., Karim S.A., Morton J.P., Del Rio Hernandez A. (2017). Matrix stiffness induces epithelial-mesenchymal transition and promotes chemoresistance in pancreatic cancer cells. Oncogenesis.

[94] Bissell M.J., Hines W.C. (2011). Why don’t we get more cancer? A proposed role of the microenvironment in restraining cancer progression. Nat. Med..

[95] Meng Z., Qiu Y., Lin K.C., Kumar A., Placone J.K., Fang C., Wang K.C., Lu S., Pan M., Hong A.W. (2018). RAP2 mediates mechanoresponses of the Hippo pathway. Nature.

[96] Chang L., Azzolin L., Di Biagio D., Zanconato F., Battilana G., Lucon Xiccato R., Aragona M., Giulitti S., Panciera T., Gandin A. (2018). The SWI/SNF complex is a mechanoregulated inhibitor of YAP and TAZ. Nature.

[97] Goetz J.G. (2009). Bidirectional control of the inner dynamics of focal adhesions promotes cell migration. Cell Adhes. Migr..

[98] Geiger B., Spatz J.P., Bershadsky A.D. (2009). Environmental sensing through focal adhesions. Nat. Rev. Mol. Cell Biol..

[99] Bershadsky A.D., Ballestrem C., Carramusa L., Zilberman Y., Gilquin B., Khochbin S., Alexandrova A.Y., Verkhovsky A.B., Shemesh T., Kozlov M.M. (2006). Assembly and mechanosensory function of focal adhesions: Experiments and models. Eur. J. Cell Biol..

[100] Canel M., Serrels A., Frame M.C., Brunton V.G. (2013). E-cadherin-integrin crosstalk in cancer invasion and metastasis. J. Cell Sci..

[101] Hynes R.O. (2002). Integrins: Bidirectional, allosteric signaling machines. Cell.

[102] Gkretsi V., Stylianopoulos T. (2018). Cell Adhesion and Matrix Stiffness: Coordinating Cancer Cell Invasion and Metastasis. Front. Oncol..

[103] Sharif G.M., Wellstein A. (2015). Cell density regulates cancer metastasis via the Hippo pathway. Future Oncol..

[104] Moroishi T., Hansen C.G., Guan K.L. (2015). The emerging roles of YAP and TAZ in cancer. Nat. Rev. Cancer.

[105] Hanahan D., Weinberg R.A. (2000). The hallmarks of cancer. Cell.

[106] Zhang J., Zhou Y., Huang T., Wu F., Pan Y., Dong Y., Wang Y., Chan A.K.Y., Liu L., Kwan J.S.H. (2019). FGF18, a prominent player in FGF signaling, promotes gastric tumorigenesis through autocrine manner and is negatively regulated by miR-590-5p. Oncogene.

[107] Ou C., Sun Z., Li X., Ren W., Qin Z., Zhang X., Yuan W., Wang J., Yu W., Zhang S. (2017). MiR-590-5p, a density-sensitive microRNA, inhibits tumorigenesis by targeting YAP1 in colorectal cancer. Cancer Lett..

[108] Sharif G.M., Schmidt M.O., Yi C., Hu Z., Haddad B.R., Glasgow E., Riegel A.T., Wellstein A. (2015). Cell growth density modulates cancer cell vascular invasion via Hippo pathway activity and CXCR2 signaling. Oncogene.

[109] Qiao Y., Chen J., Lim Y.B., Finch-Edmondson M.L., Seshachalam V.P., Qin L., Jiang T., Low B.C., Singh H., Lim C.T. (2017). YAP Regulates Actin Dynamics through ARHGAP29 and Promotes Metastasis. Cell Rep..

[110] Huang Q., Hu X., He W., Zhao Y., Hao S., Wu Q., Li S., Zhang S., Shi M. (2018). Fluid shear stress and tumor metastasis. Am. J. Cancer Res..

[111] Arslan F., Bosserhoff A.K., Nickl-Jockschat T., Doerfelt A., Bogdahn U., Hau P. (2007). The role of versican isoforms V0/V1 in glioma migration mediated by transforming growth factor-β2. Br. J. Cancer.

[112] Dulyaninova N.G., House R.P., Betapudi V., Bresnick A.R. (2007). Myosin-IIA heavy-chain phosphorylation regulates the motility of MDA-MB-231 carcinoma cells. Mol. Biol. Cell.

[113] Li Z., Xu X., Bai L., Chen W., Lin Y. (2011). Epidermal growth factor receptor-mediated tissue transglutaminase overexpression couples acquired tumor necrosis factor-related apoptosis-inducing ligand resistance and migration through c-FLIP and MMP-9 proteins in lung cancer cells. J. Biol. Chem..

[114] Kumar S., Weaver V.M. (2009). Mechanics, malignancy, and metastasis: The force journey of a tumor cell. Cancer Metastasis Rev..

[115] Jang M., Koh I., Lee J.E., Lim J.Y., Cheong J.H., Kim P. (2018). Increased extracellular matrix density disrupts E-cadherin/β-catenin complex in gastric cancer cells. Biomater. Sci..

[116] Wehrle-Haller B. (2012). Structure and function of focal adhesions. Curr. Opin. Cell Biol..

[117] Kim N.G., Gumbiner B.M. (2015). Adhesion to fibronectin regulates Hippo signaling via the FAK-Src-PI3K pathway. J. Cell Biol..

[118] Lamar J.M., Xiao Y., Norton E., Jiang Z.G., Gerhard G.M., Kooner S., Warren J.S.A., Hynes R.O. (2019). SRC tyrosine kinase activates the YAP/TAZ axis and thereby drives tumor growth and metastasis. J. Biol. Chem..

[119] Elbediwy A., Vanyai H., Diaz-de-la-Loza M.D., Frith D., Snijders A.P., Thompson B.J. (2018). Enigma proteins regulate YAP mechanotransduction. J. Cell Sci..

[120] Aragona M., Panciera T., Manfrin A., Giulitti S., Michielin F., Elvassore N., Dupont S., Piccolo S. (2013). A mechanical checkpoint controls multicellular growth through YAP/TAZ regulation by actin-processing factors. Cell.

[121] Yu F.X., Zhang Y., Park H.W., Jewell J.L., Chen Q., Deng Y., Pan D., Taylor S.S., Lai Z.C., Guan K.L. (2013). Protein kinase A activates the Hippo pathway to modulate cell proliferation and differentiation. Genes Dev..

[122] Yu F.X., Zhao B., Panupinthu N., Jewell J.L., Lian I., Wang L.H., Zhao J., Yuan H., Tumaneng K., Li H. (2012). Regulation of the Hippo-YAP pathway by G-protein-coupled receptor signaling. Cell.

[123] Feng X., Degese M.S., Iglesias-Bartolome R., Vaque J.P., Molinolo A.A., Rodrigues M., Zaidi M.R., Ksander B.R., Merlino G., Sodhi A. (2014). Hippo-independent activation of YAP by the GNAQ uveal melanoma oncogene through a trio-regulated rho GTPase signaling circuitry. Cancer Cell.

[124] Gaspar P., Tapon N. (2014). Sensing the local environment: Actin architecture and Hippo signalling. Curr. Opin. Cell Biol..

[125] Murrell M., Oakes P.W., Lenz M., Gardel M.L. (2015). Forcing cells into shape: The mechanics of actomyosin contractility. Nat. Rev. Mol. Cell Biol..

[126] Takai Y., Sasaki T., Matozaki T. (2001). Small GTP-binding proteins. Physiol. Rev..

[127] Ridley A.J. (2001). Rho GTPases and cell migration. J. Cell Sci..

[128] Timpson P., Jones G.E., Frame M.C., Brunton V.G. (2001). Coordination of cell polarization and migration by the Rho family GTPases requires Src tyrosine kinase activity. Curr. Biol..

[129] Heasman S.J., Ridley A.J. (2008). Mammalian Rho GTPases: New insights into their functions from in vivo studies. Nat. Rev. Mol. Cell Biol..

[130] Pan Y., Bi F., Liu N., Xue Y., Yao X., Zheng Y., Fan D. (2004). Expression of seven main Rho family members in gastric carcinoma. Biochem. Biophys. Res. Commun..

[131] Ridley A.J. (2016). Anne Ridley: Networking with Rho GTPases. Trends Cell Biol..

[132] Matsuoka T., Yashiro M., Kato Y., Shinto O., Kashiwagi S., Hirakawa K. (2011). RhoA/ROCK signaling mediates plasticity of scirrhous gastric carcinoma motility. Clin. Exp. Metastasis.

[133] Kakiuchi M., Nishizawa T., Ueda H., Gotoh K., Tanaka A., Hayashi A., Yamamoto S., Tatsuno K., Katoh H., Watanabe Y. (2014). Recurrent gain-of-function mutations of RHOA in diffuse-type gastric carcinoma. Nat. Genet..

[134] Sit S.T., Manser E. (2011). Rho GTPases and their role in organizing the actin cytoskeleton. J. Cell Sci..

[135] Zhao B., Li L., Wang L., Wang C.Y., Yu J., Guan K.L. (2012). Cell detachment activates the Hippo pathway via cytoskeleton reorganization to induce anoikis. Genes Dev..

[136] Yamaguchi H., Condeelis J. (2007). Regulation of the actin cytoskeleton in cancer cell migration and invasion. Biochim. Biophys. Acta.

[137] Huang T., Zhou Y., Zhang J., Wong C.C., Li W., Kwan J.S.H., Yang R., Chan A.K.Y., Dong Y., Wu F. (2017). SRGAP1, a crucial target of miR-340 and miR-124, functions as a potential oncogene in gastric tumorigenesis. Oncogene.

[138] Pan Y., Tong J.H.M., Lung R.W.M., Kang W., Kwan J.S.H., Chak W.P., Tin K.Y., Chung L.Y., Wu F., Ng S.S.M. (2018). RASAL2 promotes tumor progression through LATS2/YAP1 axis of hippo signaling pathway in colorectal cancer. Mol. Cancer.

[139] Kang M.H., Jeong G.S., Smoot D.T., Ashktorab H., Hwang C.M., Kim B.S., Kim H.S., Park Y.Y. (2017). Verteporfin inhibits gastric cancer cell growth by suppressing adhesion molecule FAT1. Oncotarget.

[140] Park H.W., Guan K.L. (2013). Regulation of the Hippo pathway and implications for anticancer drug development. Trends Pharmacol. Sci..

